# Quantum-enhanced coherent Raman spectroscopy with broadband squeezed-light detection

**DOI:** 10.1073/pnas.2611941123

**Published:** 2026-09-08

**Authors:** Konstantin E. Dorfman, Vladislav V. Yakovlev, Shaul Mukamel

**Affiliations:** ^a^Center for Theoretical Physics and School of Physics and Optoelectronic Engineering, Hainan University, Haikou 570228, China; ^b^Himalayan Institute for Advanced Study, Unit of Gopinath Seva Foundation, Rishikesh, Uttarakhand 249201, India; ^c^ Institute for Quantum Science and Engineering, Texas A&M University, College Station, TX 77843; ^d^ Department of Physics and Astronomy, Texas A&M University, College Station, TX 77843; ^e^ Department of Electrical and Computer Engineering, Texas A&M University, College Station, TX 77843; ^f^ Department of Chemistry, University of California, Irvine, CA 92697-2025; ^g^ Department of Physics and Astronomy, University of California, Irvine, CA 92697-2025

**Keywords:** quantum spectroscopy, ultrafast Raman spectroscopy, quantum squeezing

## Abstract

Quantum correlations offer the possibility of surpassing classical limits in optical measurements, but their integration into nonlinear vibrational spectroscopy has remained largely unexplored. Here, we introduce a quantum-enhanced implementation of coherent Raman spectroscopy that employs broadband squeezed-light detection to interrogate relative intensity correlations between Stokes and anti-Stokes fields generated in an ultrafast four-wave-mixing process. By exploiting nonclassical correlations between these fields, we suppress the nonresonant background and enhance the signal-to-noise performance in both temporal and spectral domains, enabling detection sensitivities beyond the classical shot-noise limit. Importantly, the quantum advantage demonstrated here does not arise solely from improved detection statistics. Rather, it originates from microscopic intermolecular correlations mediated by cascaded exchange of a virtual photon between molecular pairs. This mechanism establishes a fundamentally distinct and significant pathway for information extraction in vibrational spectroscopy, providing access to structural and dynamical observables that are intrinsically inaccessible by conventional classical Raman techniques. Numerical simulations illustrate the power of the technique by resolving intradimer C=C bond splitting in cyclobutane thymine dimers following ultraviolet excitation, highlighting its potential for probing ultrafast photochemical processes with quantum-limited sensitivity and for label-free biomedical optical sensing and imaging.

## Significance

Coherent Raman spectroscopy enables label-free chemical imaging and the study of ultrafast molecular dynamics, but its performance is fundamentally limited by nonresonant backgrounds that can obscure weak vibrational signals. Quantum correlations between broadband Stokes and anti-Stokes fields provide a powerful means to overcome this limitation. By combining ultrafast stimulated Raman excitation with homodyne detection of squeezed-light intensity correlations, nonresonant backgrounds are strongly suppressed, yielding signal-to-noise improvements of tens of decibels. This quantum-enhanced approach extends Raman spectroscopy beyond classical sensitivity limits, revealing subtle molecular structures and dynamics that are otherwise inaccessible and opening new opportunities for quantum-enabled, label-free imaging and sensing in biology, chemistry, and materials science.

Quantum light spectroscopy, sensing, and imaging have attracted substantial interest owing to their enhanced sensitivity and distinct spectral–temporal properties (1–3), which enable label-free, noninvasive interrogation of biological systems at low light intensities (4, 5). Beyond its practical applications in advanced microscopic imaging (6–10), the use of quantum light further challenges the fundamental understanding of nonlinear light–matter interactions by revealing the nature of nonclassical response functions and providing microscopic structural and dynamical information that is inaccessible with classical light (11, 12). Recent work has demonstrated that relative intensity squeezing generated via four-wave mixing in atomic vapors can substantially improve the signal-to-noise ratio in two-dimensional electronic spectroscopy of AC-Stark-shifted rubidium gas (13). Squeezed-light sources have also shown promise for subcellular biological imaging (9) and Brillouin spectroscopy by substantially improving signal-to-noise performance at low optical intensities (8).

Coherent anti-Stokes Raman spectroscopy (CARS) based on four-wave mixing (FWM) technique (14, 15) and its ultrafast version (16, 17) is a gold standard for investigating nuclear wavepacket motions on both the electronically excited and the ground states of molecules achieving both high spectral and fs temporal resolution. Moreover, in certain systems, the simultaneous detection of Stokes and anti-Stokes component may give rise to quantum correlations due to the shared phonon (18, 19) which increase with decreasing laser power, making it suitable for the detection of fragile biological samples. Femtosecond stimulated Raman spectroscopy (FSRS), is an advanced stimulated Raman technique, that provides enhanced molecular specificity by interrogating excited-state dynamics (20, 21). The technique employs a tailored pulse sequence consisting of an off-resonant picosecond pump and a femtosecond Stokes probe, which together monitor the Raman response associated with molecular rearrangements. By recording spectra as a function of pump–probe delay, FSRS enables chemical and structural measurements with both high spectral and high temporal resolution (22).

A decade ago, we developed an intuitive framework for interpreting classical FSRS signals based on a loop-diagram representation (23) and its quantum-field version (24). Within this approach, the molecular response is expressed in terms of multipoint correlation functions that can be readily obtained from microscopic quantum simulations, while field correlations are neglected, yielding the semiclassical description of the signal.

Here we introduce a quantum-enhanced coherent Raman spectroscopy (QECRS), that provides simultaneous time- and frequency-resolved access to photoinduced molecular dynamics by combining advances in both CARS and FSRS techniques. In QECRS, molecular motion is initiated by a short actinic pulse and subsequently interrogated by a sequence of pump, Stokes, and anti-Stokes pulses in a box geometry. By mixing the signal fields with a local oscillator pump and performing balanced homodyne subtraction on the output quadratures (25, 26) one can reveal quantum squeezing between Stokes and anti-Stokes fields, a broadband version of the two-mode squeezing generated in FWM shown earlier in Rb vapor (13) enabling a substantial enhancement in signal-to-noise ratio through suppression of the nonresonant background below the classical shot-noise limit that constrains conventional FSRS and CARS signals (27). We apply the QECRS to the photoreactivation dynamics of carbon–carbon bond splitting in cyclobutane thymine dimers, a critical step in the repair of UV damage of DNA, which is of central importance to life sciences and clinical applications.

**Fig. 1.**
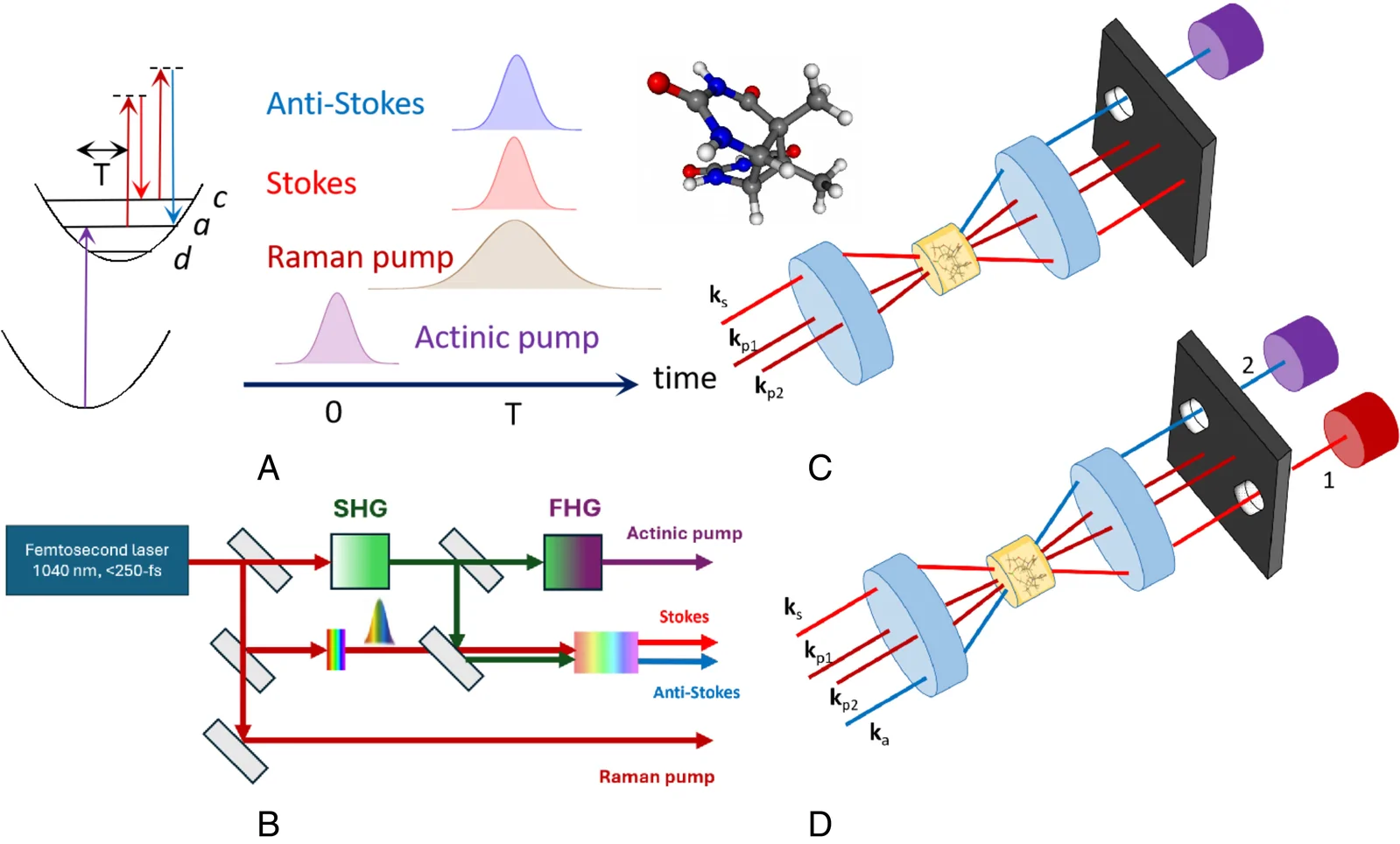
(*A*) Energy level diagram for the CRS (CARS plus CSRS) signal (*Left*) along with time-domain pulse configuration (*Middle*) and molecular structure of cyclobutane thymine dimer (*Top Right*). An actinic pulse initiates the excited state dynamics in a molecular vibrational state a. After a delay T an electronically off-resonant combination of narrowband pump and a broadband Stokes and anti-Stokes pulses captures the ultrafast dynamics giving rise to FWM transitioning system into the vibrational states c and d, respectively. (*B*) A proposed light source preparation for QECRS. A fraction of the fundamental output of a high-repetition-rate femtosecond laser (*Materials and Methods*) is frequency-doubled to 520 nm and subsequently frequency-quadrupled to produce actinic excitation pulses at 260 nm. The rest of the 520 nm radiation is used to pump an optical parametric amplifier (OPA) seeded by a white-light continuum, providing tunable signal and idler pulses, serving as the anti-Stokes and Stokes fields, respectively. The residual 1,040 nm radiation, after spectral filtering, is shaped into a narrowband picosecond Raman pump pulse. (*C*) The actinic, Raman pump, Stokes, and anti-Stokes pulses are combined using spectral beamsplitters and focused into a common interaction volume using a high–numerical aperture objective in a Box-CARS geometry. (*D*) Schematic of the proposed QECRS detection setup where the correlations between Stokes and anti-Stokes pulses propagating in the two spatial modes are detected in homodyne subtraction measurement.

The schematic of the light–matter interaction corresponding to excited state CARS or CSRS along with pulse configuration is shown in Fig. 1*A*. After the actinic pulse creates a vibrational wavepacket in excited electronic state a initiating a photophysical dynamics a delayed FWM process consisting of two pump, the Stokes or anti-Stokes pulses probe vibrational coherences ac and ad, respectively where $\omega_{c}>\omega_{a}>\omega_{d}$. Similar to FSRS the actinic, Stokes and anti-Stokes pulses are broadband, while the Raman pump pulses are narrowband. The proposed light source for QECRS is shown in Fig. 1*B* (*Materials and Methods*). Fig. 1*C* shows classical CARS process in the box geometry where the pump, and the Stokes seeding the FWM process generates anti-Stokes pulse. In contrast, in QECRS the FWM process involves interaction of two pump pulses and a pair of broadband quantized Stokes and anti-Stokes pulses, as shown in Fig. 1*D*. The Stokes pulse seeds the process of generation of the anti-Stokes pulse and vice versa, both sharing the same vibrational modes, which enhance their mutual correlation. By properly choosing two nondegenerate pump pulses, one can ensure that competing processes such as FSRS generated from the same pump pulses are ruled out. The phase-matching condition for the FWM is written as $k_{s}+k_{a}=k_{p1}+k_{p2}$. This type of double-coherent state input has been demonstrated to increase the sensitivity in SU(1,1) interferometers (28).

The quantum state of the Stoke and anti-Stokes fields is represented by a weak coherent state with average photon number $N_{s,a0}\left(\omega \right)$. The frequency-dispersed detection of the photon numbers by detectors 1 and 2 is governed by $\left\langle {\hat{N}}_{1}\left(\omega ,T\right)\right\rangle$($\left\langle {\hat{N}}_{2}\left(\omega ,T\right)\right\rangle$) at frequency ω captures Raman information due to narrowband pump pulse envelope displayed by the frequency difference $\omega -\omega_{p}$, while the delay relative to actinic pulse T provides the temporal evolution of the photophysical dynamics after the actinic pulse. The use of classical actinic pulse allows investigating photo-induced complex molecular dynamics, which occurs in the excited electronic states of molecules (22), but is not essential for the quantum correlations between Stokes and anti-Stokes discussed below. One can further detect the higher-order molecular quantities by the balanced homodyne subtraction measurement originating from the noise figure $Var({\hat{N}}_{1}\left(\omega ,T\right)-{\hat{N}}_{2}\left(\omega ,T\right))$. Clearly, the subtraction measurement must ensure that the detected photon numbers in Stokes and anti-Stokes are of the same order. On the other hand, it is well known that the corresponding cross-sections are usually very different, with the anti-Stokes one being much larger. This can be mitigated by adding both Stokes and anti-Stokes pulses for each spatial modes in the box geometry and balancing the input as shown in *SI Appendix*, Fig. S1 and the corresponding general signal calculation in *SI Appendix*, section S2. For simplicity in the following we assume similar cross-sections and adopt the simpler setup of Fig. 1*D*. Similar to two-mode squeezing generation in FWM (29), the dominant contribution to the signal originate from a pair of molecules $∼|\chi^{\left(3\right)}{|}^{2}$ (*SI Appendix*, section S1) where each molecules contributes via its $\chi^{\left(3\right)}$ susceptibility. Thus, in a weak squeezing regime the correlation function of detected Stokes and anti-Stokes fields in the QECRS signal originating from a pair of molecules is given by Eq. **3**, which is a convolution of a combined two-molecular response function in Eq. **4** and the field correlation function in Eq. **5** (*Materials and Methods*). Note, that under off-resonant excitation, response functions factorize out and the variance is governed by $Var\left({\hat{N}}_{1}-{\hat{N}}_{2}\right)∼N_{s0}\left(\omega \right)-N_{a0}\left(\omega \right)$ which vanishes under balanced condition $N_{s0}=N_{a0}=N_{0}$. This background reduction which is typical for two-mode squeezing also appears in a broadband case. On the other hand, the classical variance is governed by the photon number alone: $Var{\left({\hat{N}}_{1}-{\hat{N}}_{2}\right)}_{\mathrm{cl}}\equiv \left\langle {\hat{N}}_{1}\right\rangle +\left\langle {\hat{N}}_{2}\right\rangle$ and does not vanish giving rise to classical shot noise limit (SNL) (30). Quantum correlations suppress the nonresonant background below the SNL, thereby enhancing Raman sensitivity through an increased SNR. This enhancement arises because the molecular system can access nonclassical field correlations only when the correlated Stokes and anti-Stokes fields temporally overlap. The exchange of quantum correlations between the light field and the molecular system occurs on the timescale of vibrational dephasing, which in the proposed scheme is comparable to the pulse duration. Consequently, purely time-domain measurements, such as transient grating and conventional time-resolved stimulated Raman experiments, where Stokes and anti-Stokes pulses are applied in a certain time sequence, do not effectively access these quantum correlations, particularly, in the long-dephasing regime. Such configurations could, however, be adapted by engineering both Stokes and anti-Stokes fields to contribute to the same spatial modes, thereby restoring temporal overlap; this extension will be explored in future work.

**Fig. 2.**
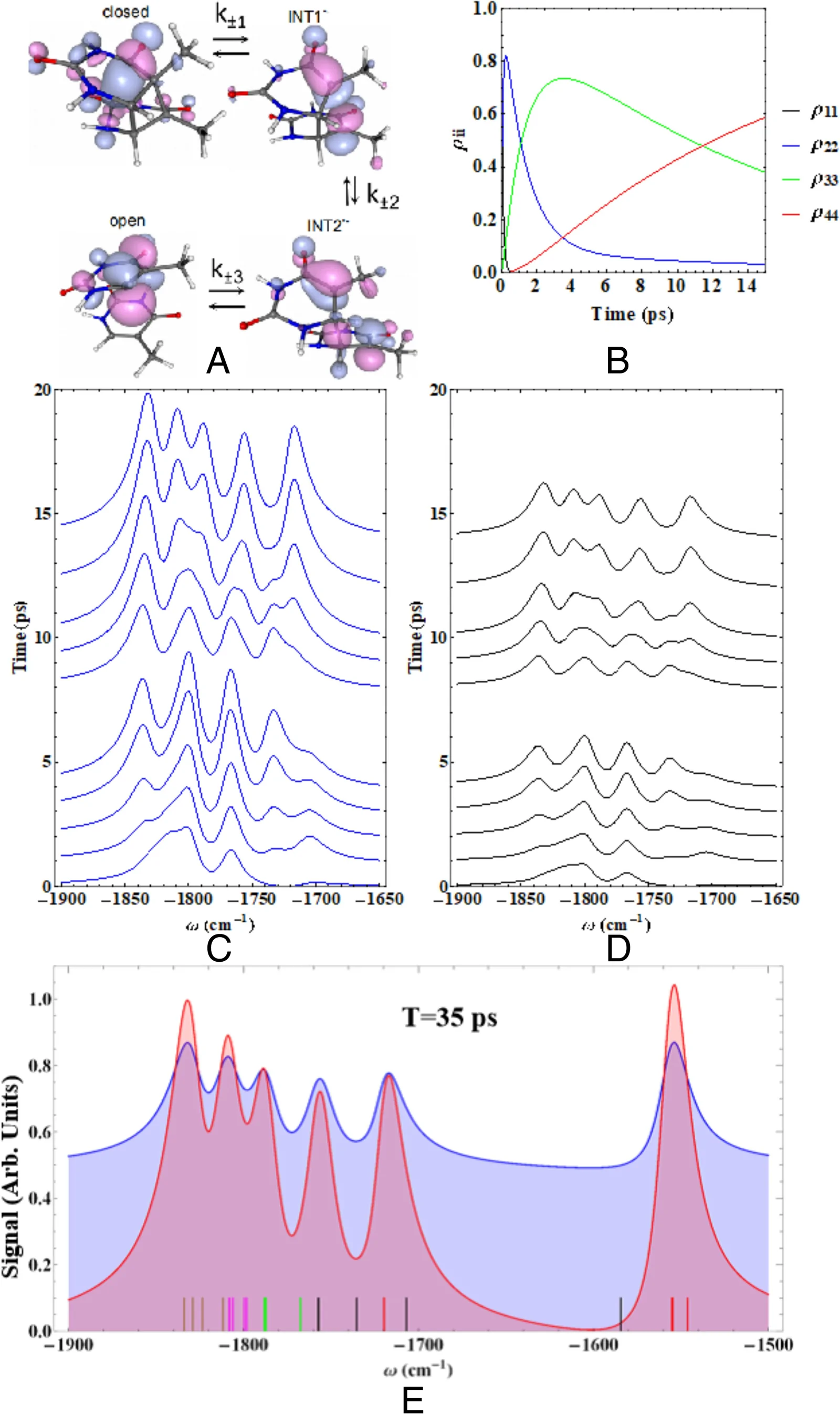
(*A*) Vibrational modes conformations connected by forward and backward population transfer in four-state jump (FSJ) model. (*B*) the excited state FSJ dynamics between “closed” and “open” configuration in the C=C bond splitting dynamics for the cyclobutane thymine molecule. (*C* and *D*) The series of snapshot Raman spectra for the QECRS signal ranging from T=0 and T=35 ps with quantum squeezing and classical detection, respectively. (*E*) The snapshot of the Raman spectra showing the suppressed nonresonant background of the “squeezed” spectrum at T=35 ps. Colorful stick spectra correspond to five vibrational states each undergoing FSJ kinetics. The signal has been normalized at a peak near 1,720 cm^−1^, corresponding to eigenvalue of the intermediate state 2 of the bond splitting process.

We now compare classical CRS (CARS and CSRS) and quantum QECRS signals for the $C_{6}=C_{6}$ bond splitting process in a cyclobutane thymine molecule. Experiments (31) show that this process is governed by the sequential mechanism where the molecule undergoes rearrangement from closed form to two intermediate steps INT1, INT2, and open form denoted by the populations $\rho_{11}$, $\rho_{22}$, $\rho_{33}$, and $\rho_{44}$ in Fig. 2 *A* and *C*. The splitting dynamics is described by the rate equation Eq. **7** and the corresponding population dynamics is shown in Fig. 2*B*. We find that around 67.5 fs the population of closed and INT1 states $\rho_{11}$ and $\rho_{22}$ become equal with $\rho_{22}$ reaching maximum around 226 fs. Population of two INT states further equilibrate at 1.1 ps with population $\rho_{33}$ reaching maximum around 3.5 ps. Finally the equilibration between INT2 and open form is reached at around 11.4 ps and final population transfer to open form $\rho_{44}$ is reached at 45 ps. The calculated forward rates $k_{1,2,3}$ are based on the experimental work by Liu et al. (31) and are an order of magnitude faster than the corresponding back reaction rates $k_{-1,-2,-3}$. The parameters used in the following simulations are listed in ref. 32. Fig. 2*C* and d depict the QECRS and classical CRS signal as a series of snapshots of Raman spectra governed by *SI Appendix*, Eq. **S21**. Due to the homodyne nature of the signal the dispersive lineshapes at T<100 fs normally observed in FSRS signal are not present. On the other hand the absorptive peaks follow the transient geometries according to the slow sequential nature of the SLE-governed kinetics. The signals can thus closely monitor the bond rearrangement. During the first 4 ps, four peaks emerge in the range of 1,730 to 1,900 cm^−1^, which corresponds to the completion of $C_{5}=C_{5}$ splitting with dominant contribution from INT2 state $\rho_{33}$. During the next 16 ps, spectra contain five peaks at 1,700 to 1,900 cm^−1^, which corresponds to $C_{6}=C_{6}$ splitting dominated by the open form $\rho_{44}$. The comparison of classical CRS and QECRS shows higher contrast of QECRS due to reduction in the off-resonant background between the resonant peaks. Fig. 2*E* compares the snapshot at T=35 ps showing significant reduction in the off-resonant background by several orders of magnitude especially in the area between 1,600 to 1,700 cm^−1^. Thus the squeezing significantly reduces the spectral congestion and increases the SNR. To improve the SNR one can increase an intensity of one of the pulses, which may increase the signal at a particular frequency range. The downside of such an approach is the sensitivity of biomolecules to high intensity radiation and low photodamage threshold. In the opposite low intensity regime, which is more biologically suitable, the peak intensity of Raman spectra will be hardly above the SNL.

To further investigate the difference between QECRS and CRS we introduce the two-molecule component of quantum advantage (QA) (33) that characterizes the degree of relative squeezing of the Stokes and anti-Stokes fields relative to classical noise level.

**Fig. 3.**
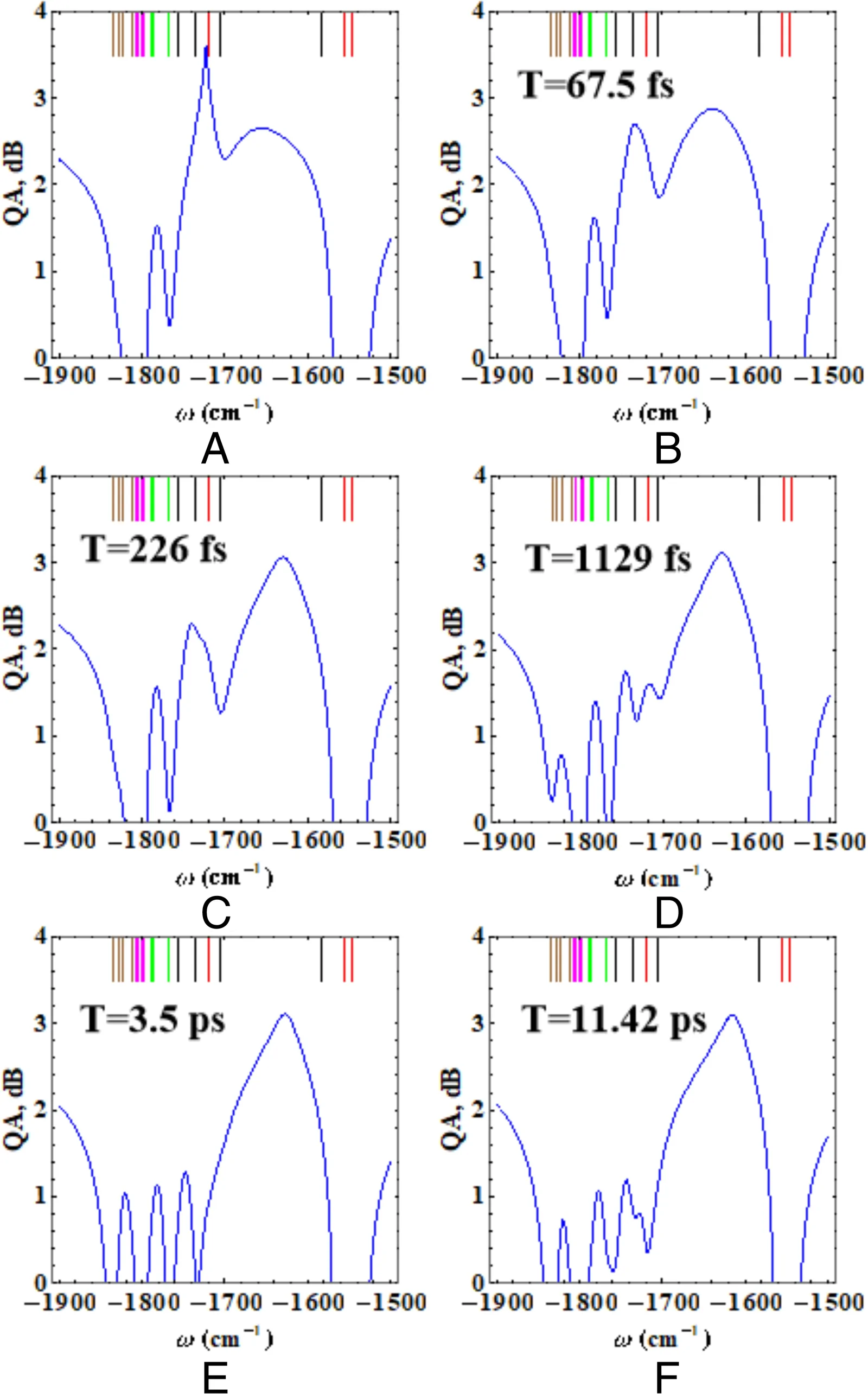
Quantum advantage in Eq. **1** vs. the Raman frequency shift at different delays T=1 fs, 67.5 fs, 226 fs, 1129 fs, 3.5 ps, and 11.42 ps correponding to panels (*A–F*), respectively. Colorful stick spectra correspond to five vibrational states each undergoing FSJ kinetics. The rest of parameters used for simulations are same as in Fig. 2.

**Fig. 4.**
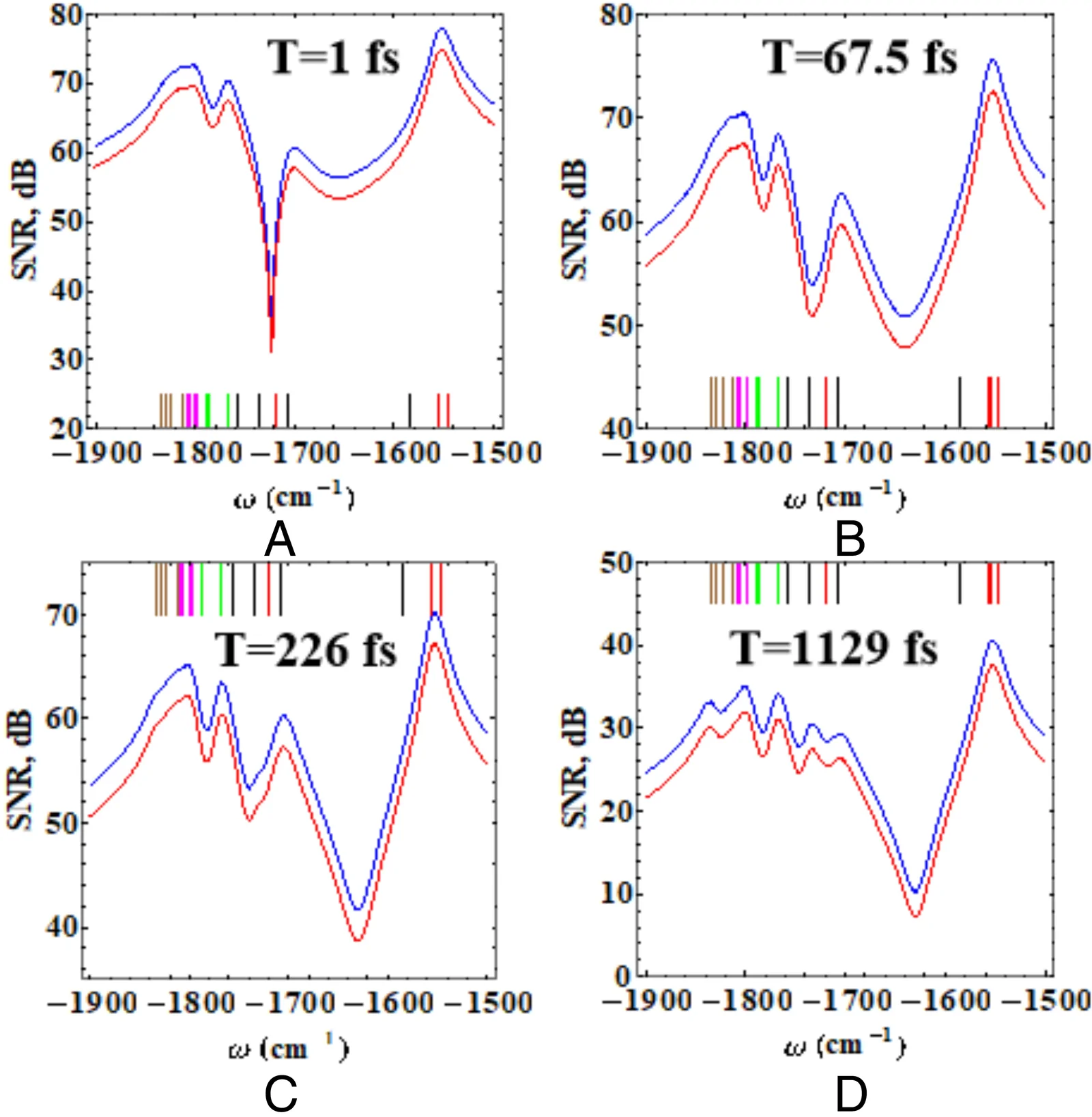
SNR in Eq. **2** for QECRS with weak coherent Stokes and anti-Stokes pulses—blue, and CRS with classical pulses—red at different delay times T. T=1 fs, 67.5 fs, 226 fs, and 1129 fs corresponding to panels (*A–D*), respectively. Colorful stick spectra correspond to five vibrational states each undergoing FSJ kinetics. The rest of parameters used for simulations are same as in Fig. 2.

[1] $$\begin{array}{c} QA_{2}\left(\omega ,T\right)=\frac{\text{Var}{\left({\hat{N}}_{1}\left(\omega ,T\right)-{\hat{N}}_{2}\left(\omega ,T\right)\right)}_{2}}{\text{Var}{\left({\hat{N}}_{1}\left(\omega ,T\right)-{\hat{N}}_{2}\left(\omega ,T\right)\right)}_{2Cl}}, \end{array}$$

where $\text{Var}{\left({\hat{N}}_{1}\left(\omega ,T\right)-{\hat{N}}_{2}\left(\omega ,T\right)\right)}_{2Cl}={\left\langle {\hat{N}}_{1}\left(\omega ,T\right)\right\rangle }_{2}+{\left\langle {\hat{N}}_{2}\left(\omega ,T\right)\right\rangle }_{2}$. Fig. 3*A*–*F* depicts the Raman spectra snapshots from T=1 fs to T=11.42 ps for $QA_{2}$ corresponding to the times relevant to population equilibration and transfer discussed in previous section and shown in Fig. 2*B*. It shows gradual diminishing of the QA with time in the range of 1,700 to 1,800 cm^−1^ from 3.5 dB down to 1 dB. The weakening signal at longer interpulse delays makes it more difficult to maintain consistent squeezing and consequently reduces off-resonant background in the highly congested spectral range. On the other hand the range 1,600 to 1,700 cm^−1^ contains sparse spectral peaks and the corresponding background reduction remains strong and unaffected by signal decay reaching ∼3 dB. To further quantify the reduced noise level we plot SNR for one of the detectors (8) shown in Fig. 4 given by

[2] $$\begin{array}{c} SNR_{2}\left(\omega ,T\right)=\frac{{\left\langle {\hat{N}}_{1}\left(\omega ,T\right)\right\rangle }_{2}}{{\left(\text{Var}{\left({\hat{N}}_{1}\left(\omega ,T\right)\right)}_{2}\right)}^{1/2}}. \end{array}$$

The series of snapshots from 1 fs to 1.1 ps show the stable enhancement of SNR between classical CRS and QECRS in the range of ∼2 to 3 dB, consistent with the QA in Fig. 3 and similar experimental setups (34). Relatively high overall SNR ∼60 to 70 dB indicates absence of losses which are not included in the model but can be easily incorporated within input–output relations (35). Moreover, the strategies for minimizing those losses are well established, and, for example, described in ref. 36. In brief, specially designed microscope objectives are optimized by high-throughput (low-loss) beam transmission, while detectors are chosen to maximize the quantum efficiency. Since, with some modifications in excitation and detection arrangement, the setup resembles a typical CARS/SRS used for microscopic imaging (37) with the obvious advantage of improved SNR and spectral resolution of such imaging at the same level of used laser power, which lead to improved sensitivity and specificity of detection, respectively.

In summary, we predict a frequency-dispersed QECRS signals with multimode quantum light fields in a low-to-moderate squeezing regime. The squeezing, generated in the course of FWM reduces the nonresonant background below the classical shot noise limit contributing to higher signal-to-noise ratio and higher sensitivity of quantum Raman signals at low intensity regime. Note, that other features of the squeezed signals such as enhanced resolution can be also demonstrated for this process but require a different set of signals and microscopic details of field–matter interactions similar to two-photon absorption (38). An additional quantum spectroscopy feature is related to nonclassical response function which is generally different from semiclassical response function used in this work. This effect originates from noncausal response function manifest as a microscopic cascading effect in the two-molecule exchange of a virtual photon which manifests as interchange between absorptive and dispersive lineshapes for a particular quantum states of light and has no classical analogy. To observe this effect, a single reaction pathway with two vibration modes with a $∼|R_{s}{|}^{2}$ response function and coherently seeded Stokes/anti-Stokes pulses is not sufficient, and one has to incorporate a larger number of degrees of freedom which will include two-molecular pathways of the material response function $∼|R_{a}{|}^{2}$, $∼R_{s}R_{a}^{*}$, and $R_{a}R_{s}^{*}$ and their combination accompanied by different field correlation functions, which are combined in a different form compared to semiclassical response function. As a striking example one can consider the standard FWM scheme where anti-Stokes is generated spontaneously from vacuum (13) with $N_{a0}=0$. In this case certain time-ordered pathways $∼\langle {\hat{a}}_{a}^{†}{\hat{a}}_{a}\rangle =0$ will vanish while contributions with $∼\langle {\hat{a}}_{a}{\hat{a}}_{a}^{†}\rangle =1$ will remain giving rise to a different structure of the response function. This aspect of quantum signals will be the focus of the future work and will be published elsewhere. The presented work paves the way for highly sensitive chemical biological sensors with quantum light with advanced sensitivity that can target the novel microscopic information not accessible with classical light sensing tools.

## Methods

### QECRS signal.

The QECRS can be derived by taking the field–matter interactions to fourth order with respect to ${\hat{H}}_{\mathrm{int}}$ in *SI Appendix*, Eq. **S6** reading the signal from the Feynman diagrams (*SI Appendix*, section S2) as

[3] $$\begin{array}{cc} & {\left\langle f\left[{\hat{N}}_{1},{\hat{N}}_{2}\right]\left(\omega ,T\right)\right\rangle }_{2}\propto \int_{-\infty }^{\infty }dtdt^{\prime }e^{i\omega (t-t^{\prime })}\int_{-\infty }^{t}d\tau \int_{-\infty }^{t^{\prime }}d\tau^{\prime }\times \\ & \sum_{i,j=s,a}C_{\mathrm{ij}}^{\left(f\right)}\left(\omega ,\tau ,\tau^{\prime }\right)C_{p}\left(t,t^{\prime },\tau ,\tau^{\prime }\right)R_{i}\left(t,\tau \right)R_{j}^{*}\left(t,\tau^{\prime }\right), \end{array}$$

where the pump pulse contribution is given by $C_{p}\left(t,t^{\prime },\tau ,\tau^{\prime }\right)=E_{p}\left(t\right)E_{p}^{*}\left(t^{\prime }\right)E_{p}\left(\tau \right)E_{p}^{*}\left(\tau^{\prime }\right)$ and photon number detection comes from $f=\langle {\hat{N}}_{1,2}\rangle$ while variances can be $f=Var({\hat{N}}_{1,2})$, $f=Cov({\hat{N}}_{1},{\hat{N}}_{2})$ or $f=Var({\hat{N}}_{1}-{\hat{N}}_{2})$. The molecular response function is given by

[4] $$\begin{array}{cc} & R_{s}\left(t,\tau \right)=\alpha_{\mathrm{ac}}^{2}G_{\mathrm{ac}}\left(t-\tau \right)G_{\mathrm{aa}}\left(\tau \right)\\ & R_{a}\left(t,\tau \right)=\alpha_{\mathrm{ad}}^{2}G_{\mathrm{ad}}\left(t-\tau \right)G_{\mathrm{aa}},\left(\tau \right), \end{array}$$

where $\alpha_{\mathrm{ak}}$, k=c,d is the electronically off-resonant Raman polarizability and the population and coherence Green’s functions $G_{\mathrm{aa}}$ and $G_{\mathrm{aj}}$, j=c,d are calculated using the stochastic Liouville equation (SLE) (Eq. **8** and *SI Appendix*, section S3). We focus on the negative Raman shift $\omega -\omega_{p}=-\omega_{\mathrm{ac}}$ which has a dominant contribution from the $|R_{s}{|}^{2}$ response function. More general setup with combined Stokes/anti-Stokes input is discussed in *SI Appendix*, section S2. The corresponding quantum field correlation functions are given by

[5] $$\begin{array}{c} C_{\mathrm{ss}}^{\left(N_{1}\right)}=2(2N_{a0}A_{a}^{*}\left(\tau \right)A_{a}\left(\tau^{\prime }\right)+|A_{a}\left(\tau -\tau^{\prime }\right){|}^{2}),\;C_{\mathrm{ss}}^{\left(N_{2}\right)}=0, \end{array}$$

[6] $$\begin{array}{cc} & C_{\mathrm{ss}}^{V\;ar(N_{1}-N_{2})}=4[C_{a}\left(\tau -\tau^{\prime }\right)(N_{s0}|A_{s}\left(\omega \right){|}^{2}+N_{a0}|A_{a}\left(\omega \right){|}^{2})\\ & +2N_{a0}\left[A_{a}^{*}\left(\tau \right)A_{a}\left(\tau^{\prime }\right)-{\tilde{A}}_{a}^{*}\left(\omega ,\tau \right)A_{a}\left(\tau^{\prime }\right)-A_{a}^{*}\left(\tau \right){\tilde{A}}_{a}\left(\omega ,\tau^{\prime }\right)\right], \end{array}$$

where ${\tilde{A}}_{a}\left(\omega ,t\right)=A_{a}\left(\omega \right)e^{-i\omega (t-T)}$, $A_{j}\left(t\right)=\int d\omega \;A_{a}\left(\omega \right)e^{-i\omega t}$ is a time-domain Gaussian envelope for the quantum field j=s,a.

### Experimental Setup.

To validate the proposed mechanisms for quantum-enhanced vibrational imaging, the experimental setup currently under construction at TAMU is designed as follows. A high-repetition-rate femtosecond laser source (e.g., a diode-pumped Yb-doped fiber laser or Yb:KGW system) generates ∼250 fs pulses at MHz repetition rates, with pulse energies exceeding 10 μJ at a central wavelength of approximately 1,040 nm. The OPA is seeded by a white-light continuum generated in a 2 mm-thick YAG crystal by focusing 1 to 2 μJ of the 1,040 nm fundamental radiation. Independent optical delay lines (not shown in Fig. 1*B*) are incorporated into each beam path to enable precise temporal synchronization. In addition, combinations of waveplates and polarizers (also not shown in Fig. 1*B*) provide full control over the polarization states and relative intensities of all interacting beams. If necessary, dispersion compensation elements can be introduced in each optical path to fine-tune pulse durations and optimize temporal overlap at the interaction region.

In contrast to conventional detection schemes, two detectors are employed to enable the correlation measurements described above. These measurements can be performed as functions of both the interpulse time delays and the Raman frequency, allowing direct quantitative comparison with the theoretical predictions presented in Figs. 2–4. We note that the proposed experimental configuration is readily extendable to imaging applications by scanning the focal volume in three dimensions across the sample of interest, such as a biological cell or tissue specimen. The anticipated improvement in the SNR enables higher acquisition speeds while maintaining the noninvasive character of the measurement.Background suppression enhances image contrast while increasing the specificity and fidelity of signal detection.

### Stochastic Liouville Equation.

The multistep first-order kinetics and transition state theory implies the linear sequence of intradimer bond splitting where the initial state of the bath 1 (closed form), two intermediate states (2 and 3), and finally open form (4) are coupled by the forward rates $k_{1,2,3}$ and backward rates $k_{-1,-2,-3}$ which satisfy the rate equation

[7] $$\begin{array}{c} {\dot{\rho }}_{\mathrm{aa}}\left(t\right)=-K\rho_{\mathrm{aa}}\left(t\right), \end{array}$$

where the population $\rho_{\mathrm{aa}}\left(t\right)$ is a four-component vector including the states 1 to 4, with state $\rho_{11}\left(t=0\right)=1$ initially excited by the actinic pulse. The solution of Eq. **7** is shown in Fig. 2*B*.

QECRS signals may be calculated using the stochastic Liouville equation (23, 39), which allows to calculate spectral line shapes of a quantum system coupled to a classical bath. It assumes that the classical stochastic bath dynamics is independent from the system while the system is affected by the bath. The system plus bath density matrix evolves according to:

[8] $$\begin{array}{c} \dot{\hat{\rho }}=\hat{L}\hat{\rho }=-\frac{i}{\hbar }\left[\hat{H},\hat{\rho }\right]-K\hat{\rho }, \end{array}$$

where $\hat{H}$ is the system Hamiltonian that depends parametrically on the bath. The system has three vibrational states a, c, and d and the vibrational frequencies $\omega_{ca,s}$ and $\omega_{ad,s}$ captured by the Stokes and anti-Stokes probes are perturbed by the bath. For each pair of vibrational states (e.g. a, and c) the total density matrix ρ has 16 components$|\nu \nu^{\prime }s\rangle \rangle$ which corresponds to the direct product of four Liouville space states $|\nu \nu^{\prime }\rangle \rangle$, where $\nu ,\nu^{\prime }=a,c$, and four bath states s. The Liouville operator $\hat{L}$ is diagonal in the space of vibrational states and is given by four 4 × 4 diagonal blocks in the bath space:

[9] $$\begin{array}{c} {\left[\hat{L}\right]}_{\nu \nu^{\prime }s,\nu_{1}\nu_{1}s^{\prime }}=\delta_{\nu \nu_{1}}\delta_{\nu^{\prime }\nu_{a}^{\prime }}\left({\left[{\hat{L}}_{S}\right]}_{s,s^{\prime }}+\delta_{ss^{\prime }}{\left[\hat{L}\right]}_{\nu \nu^{\prime }s,\nu \nu^{\prime }s}\right), \end{array}$$

where ${\hat{L}}_{S}=-K$ describes the kinetics given by Eq. **8** and the coherent part ${\hat{L}}_{S}$ is given by the first term in Eq. **8** describes the vibrational dynamics (*SI Appendix*, Eqs. **S18** and **S19**). Following the approach outlined in ref. 23, we obtain the Green’s functions which enter the response function in Eq. **4** (for details see *SI Appendix*, section S3).

## Supplementary Material

Appendix 01 (PDF)

## Data Availability

All study data are included in the article and/or *SI Appendix*.
