# Supplementary material for: Quantum-enhanced coherent Raman spectroscopy with broadband squeezed-light detection
Source: Proc Natl Acad Sci U S A. 2026 Sep 8;123(37):e2611941123. doi: 10.1073/pnas.2611941123 (PMC13578947; doi:10.1073/pnas.2611941123)
Supplement: Supplementary file 1 [file pnas.2611941123.sapp.pdf]

Supplementary Information: Quantum Enhanced Coherent Raman Spectroscopy with Broadband Squeezed Light Detection

Konstantin E. Dorfman[1,2‡,*] Vladislav V. Yakovlev[3,4,5], and Shaul Mukamel[6]
[1] *Center for Theoretical Physics and School of Physics and Optoelectronic Engineering, Hainan University, Haikou 570228,China*
[2] *Himalayan Institute for Advanced Study, Unit of Gopinath Seva Foundation, MIG 38, Avas Vikas, Rishikesh, Uttarakhand 249201, India*
[3] *Department of Biomedical Engineering, Texas AM University, College Station, TX 77843, USA*
[4] *Department of Physics and Astronomy, Texas AM University, College Station, TX 77843, USA*
[5] *Department of Electrical and Computer Engineering, Texas AM University, College Station, TX 77843, USA*
[6] *Chemistry Department and Physics and Astronomy Department, University of California, Irvine, California 92697-2025, USA*
(Dated: August 10, 2026)

S1. S1. TWO-MODE SQUEEZING.

The FWM in CW regime which involves two classical (pump and probe) and two quantum (Stokes and anti-Stokes) fields that are described by classical pump amplitude $\mathscr{E}_p$ and operators for the quantum fields ($\hat{a}_s$ and $\hat{a}_a$). Initial state of the Stokes field is coherent state $\psi_s(t=0) = |\alpha\rangle$ with average photon number $|\alpha|^2 = N_{s0}$, while the anti-Stokes is generated from vacuum $\psi_a(t=0) = |0\rangle$ The phase matching for the FWM is given by: $\omega_p - \omega_s = \omega_a - \omega_p$ and $\mathbf{k}_p - \mathbf{k}_s = \mathbf{k}_a - \mathbf{k}_p$. The standard treatment of two-mode squeezing generated via FWM is described by Bogoliubov transformation which results in the input-output relation between two quantum fields [1]:

$$\begin{aligned}\hat{a}_s &\to \cosh(r)\hat{a}_s + \sinh(r)\hat{a}_a^\dagger,\\ \hat{a}_a^\dagger &\to \sinh(r)\hat{a}_s + \cosh(r)\hat{a}_a^\dagger,\end{aligned} \tag{S1}$$

where the squeezing parameter $r = A\chi^{(3)}(-\omega_s,-\omega_a;\omega_p)$ with $A \propto \mathscr{E}_p^2 L$ is the normalization constant, L is a propagation length inside the FWM media. As the result of the input-output relation described by Eq. (S1) the photon number of the Stokes and anti-Stokes fields read:

$$\begin{aligned}\langle\hat{N}_s\rangle &\simeq GN_{s0},\\ \langle\hat{N}_a\rangle &\simeq (G-1)N_{s0},\end{aligned} \tag{S2}$$

where

$$G(-\omega_s,-\omega_a;\omega_p) = \cosh^2[|A\chi^{(3)}(-\omega_s,-\omega_a;\omega_p)|] \tag{S3}$$

is the FWM gain.

Thus, by measuring the Stokes or anti-Stokes intensities one can directly detect $\chi^{(3)}$. This standard treatment does not distinguish the type of $\chi^{(3)}$ which enters Stokes and anti-Stokes fields, assuming they are the same. This assumption is typically valid in the off-resonant FWM regime.

In addition to the FWM gain one can measure QA or the noise figure of the intensity difference that characterizes the degree of relative squeezing of the probe and the conjugate fields relative to classical noise level. It is defined as the relative intensity noise divided by that of a classical beams [2]:

$$QA = \frac{\mathrm{Var}(\hat{N}_s - \hat{N}_a)}{\langle\hat{N}_s\rangle + \langle\hat{N}_a\rangle} = \frac{1}{2G(-\omega_s,-\omega_a;\omega_p)-1}, \tag{S4}$$

The above result indicates that for a sufficiently large gain $G > 1$ the noise figure always smaller than classical shot noise level $QA < 1$. This result is consequence of using Bogoliubov theory in Eq. (eq:inout) and is therefore limited to the two-mode squeezing and off-resonant FWM interaction. In the case of multi-mode field (short optical pulses) one can use theory, which is typically used in the quantum light spectroscopy, based on diagrammatic perturbative expansion. This assumption is consistent with the recent finding that most effective multi-mode squeezing [3] is achieved in low-to-moderate squeezing regime [4]. In the low squeezing regime we obtain:

$$\begin{aligned}Var(\hat{N}_s) &= (1+3|r|^2)N_{s0},\\ Var(\hat{N}_a) &= |r|^2 N_{s0},\\ CoVar(\hat{N}_s, N_a) &= 2|r|^2 N_{s0}\end{aligned} \tag{S5}$$

such that $Var(\hat{N}_s - \hat{N}_a) = N_{s0}$. Since $r \sim \chi^{(3)}$, which originates from a single molecule, the numerator in Eq. (S4) 4rhas vanishing contributions from light-matter interactions. This can be further checked in higher order terms. Therefore, to illustrate the quantum squeezing effect on the multi-mode FWM one can analyze coherent signal contribution (signal originated from a pair of molecules) for a pulsed FWM and show that the corresponding nonresonant background vanishes, which makes it below the shot-noise level, which corresponds to the denominator in Eq. (S4), which yields: $Var(\hat{N}_s - \hat{N}_a)_{SNL} = (1+2|r|^2)N_{s0}$.

S2. S2. DERIVATION OF QESRS

Here we present the calculation of the generalized quantum signal. In the traditional CARS setup two Raman

* To whom correspondence should be addressed. E-mail: dorfmank@hainanu.edu.cn, yakovlev@tamu.edu, or smukamel@uci.edu

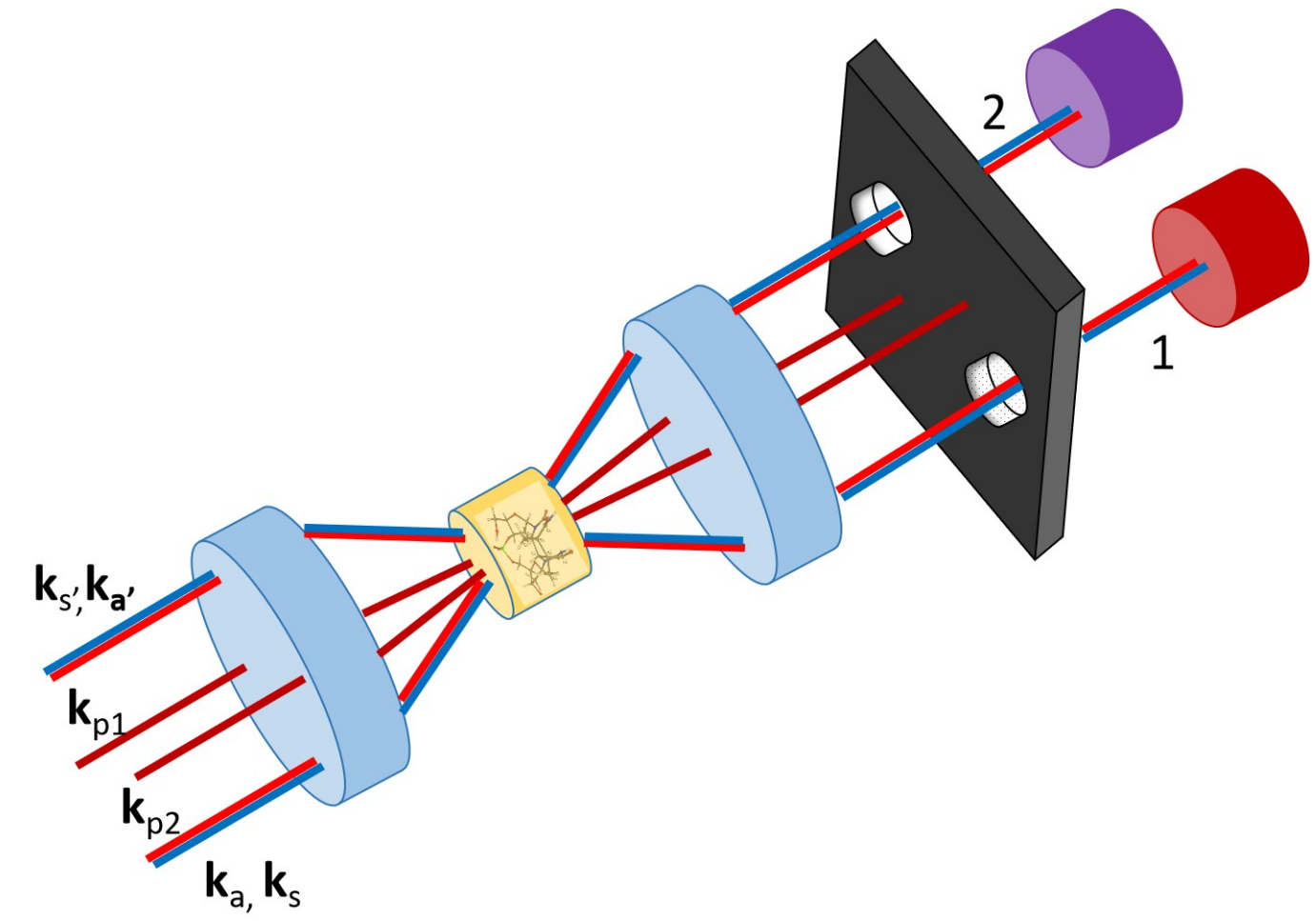

FIG. S1. Generalized detection setup from Fig. 1d of the main text. Each spatial mode contains a pair of Stokes and anti-Stokes pulses.

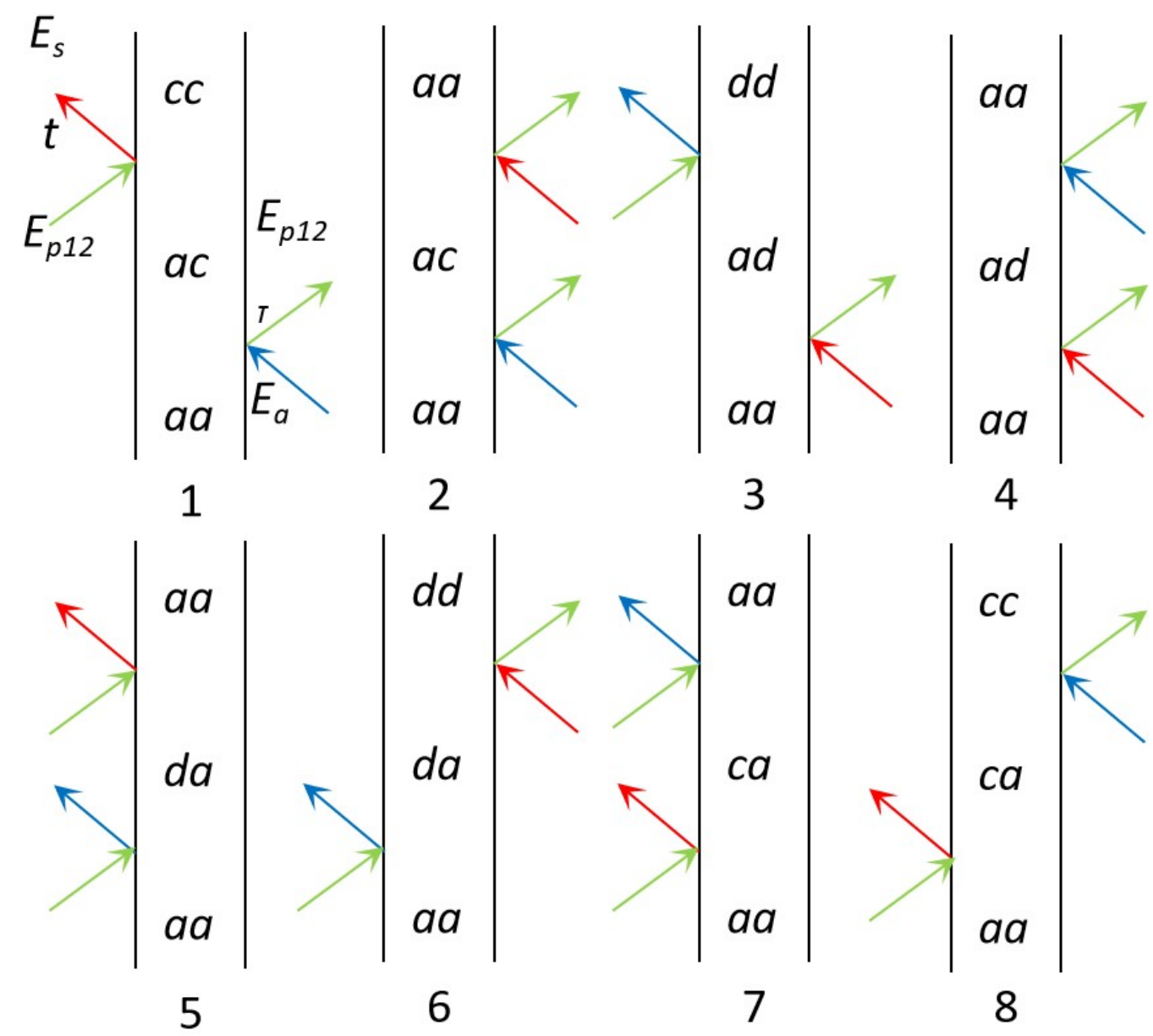

FIG. S2. Set of 8 Feynman diagrams which along with 8 conjugate diagrams (total is $8 \times 8 = 64$ terms) corresponding to QESRS signal.

pump pulses $\mathscr{E}_p(t)e^{i\mathbf{k}_{p1,2}\mathbf{r}} \simeq \mathscr{E}_p e^{-i\omega_p(t-T)+i\mathbf{k}_{p1,2}\mathbf{r}}$ and Stokes pulse $\mathscr{E}_s(t)e^{i\mathbf{k}_s\mathbf{r}} \simeq \int d\omega_s \mathscr{E}_s(\omega_s)e^{-i\omega_s(t-T)+i\mathbf{k}_s\mathbf{r}}$ generate an anti-Stokes signal measured in a frequency-dispersed detector. In the case of QECRS instead of single classical Stokes pulse, we propose to use a pair of broadband quantized Stokes and anti-Stokes pulses as shown in Fig. 1d: $\hat{E}_j(t)e^{i\mathbf{k}_j\mathbf{r}} = \int d\omega A_j(\omega)\hat{a}_j(\omega)e^{-i\omega(t-T)+i\mathbf{k}_j\mathbf{r}}$ with Gaussian envelope $A_j(\omega) = e^{-(\omega-\omega_{j0})^2/2\sigma_j^2}$ $j = s,a$. The quantized Stokes and anti-Stokes fields satisfy commutation relation $[\hat{a}_j(\omega), \hat{a}_j^\dagger(\omega')] = \delta(\omega-\omega')$. The quantum nature of these fields is represented by a weak coherent state $|\alpha_j\rangle$ with average photon number $N_{j0}(\omega) = |\alpha_j(\omega)|^2 = \langle\alpha_j|\hat{a}_j^\dagger(\omega)\hat{a}_j(\omega)|\alpha_j\rangle$ with equivalent field amplitude much smaller than of the pump field: $|\mathscr{E}_j(\omega)| \equiv |A_j(\omega)\alpha_j(\omega)| \ll |\mathscr{E}_p|$ $j = s,a$. Instead of a single pair of Stokes and anti-Stokes quantum pulses representing two spatial modes propagating along $\mathbf{k}_1$ and $\mathbf{k}_2$ directions, respectively we introduce two pair of these pulses. Combined with the balance input this allows to mitigate problem of Stokes and anti-Stokes cross-section difference in the subtraction measurement. Each spatial mode contains a pair of Stokes and anti-Stokes fields ($\hat{E}_s$, $\hat{E}_a$) and ($\hat{E}_{s'}$, $\hat{E}_{a'}$) propagating along $\mathbf{k}_1$ and $\mathbf{k}_2$, respectively (see Fig. S1). The corresponding phase matching for FWM is given by: $\mathbf{k}_s + \mathbf{k}_{a'} = \mathbf{k}_{s'} + \mathbf{k}_a = \mathbf{k}_{p1} + \mathbf{k}_{p2}$. In this case the detected fields $\hat{E}_1(\omega) = \hat{E}_{s'}(\omega) + \hat{E}_{a'}(\omega)$ and $\hat{E}_2(\omega) = \hat{E}_s(\omega) + \hat{E}_a(\omega)$ with commucation rules $[\hat{E}_j(\omega), \hat{E}_j(\omega)] = |A_j(\omega)|^2$, $j = s,a,s',a'$. This generalization is done to demonstrate that in the case of more complex system dynamics when all contributions from responses are included, i.e. $|R_s|^2$, $|R_a|^2$, $R_sR_a^*$, and $R_aR_s^*$ the reduction of the nonresonant background is still valid. Moreover, it opens the possibility to construct nonclassical response function by combining R_s and R_a pathways weighted by quantum field correlation functions. In the same time, to address the clear CRS analogue in the main text we keep only a single pair of quantum fields for brevity.

The interaction Hamiltonian for the Raman process is given by

$$\hat{H}_{int}(t) = \mathscr{E}_p(t)[\alpha_{ac}\hat{E}_s^\dagger(t) + \alpha_{ad}\hat{E}_a^\dagger(t)] + H.c., \tag{S6}$$

where α_{ak}, $k = c,d$ is an off-resonant Raman susceptibility. The two-molecule contribution to signal originate from the set of 8 diagrams shown in Fig. S2 multiplied by corresponding 8 conjugate diagrams, each stemming from fourth order in interaction Hamiltonian in Eq. (S6). Diagrams 1,2,5,6 contribute to R_s, while diagrams 3,4,7,8 are part of R_a contribution assuming $\omega_{ca} = \omega_{ad}$. While the classical CRS signals are described by diagram 1, and 3 for Stokes and 5 and 7 for anti-Stokes respectively, the quantum signals have additional contributions from diagrams 2,4,6,8, where the last interactions are with bra- rather than ket highlighting field commutators (see below). We start with the single molecule contribution to the photon numbers $\langle\hat{N}_{1,2}\rangle_1$ and which will take part in the variance through its square (see Eq. (S17) below. The total photon number is given by

$$\langle\hat{N}_1\rangle_1 = \langle\hat{N}_{s'}\rangle_1 + \langle\hat{N}_{a'}\rangle_1 + \langle\hat{E}_{s'}^\dagger\hat{E}_{a'}\rangle_1 + \langle\hat{E}_{a'}^\dagger\hat{E}_{s'}\rangle_1.$$
$$\langle\hat{N}_2\rangle_1 = \langle\hat{N}_s\rangle_1 + \langle\hat{N}_a\rangle_1 + \langle\hat{E}_s^\dagger\hat{E}_a\rangle_1 + \langle\hat{E}_a^\dagger\hat{E}_s\rangle_1. \tag{S7}$$

To calculate individual terms in Eq. (S7 we account to second order in field-matter interactions and obtain

$$\langle\hat{f}_s^{(1)}(\hat{E}_s)\hat{f}_a^{(1)}(\hat{E}_a)\rangle_1 \propto \int_{-\infty}^{\infty} dt \int_{-\infty}^{t} d\tau$$
$$\sum_{k=s,a} C_k^{(1)}(\hat{f}_s^{(1)}, \hat{f}_a^{(1)})\mathscr{E}_p(t)\mathscr{E}_p(\tau)R_k(t,\tau) + c.c., \tag{S8}$$

where $\hat{f}_j^{(1)}(\hat{E}_j)$ can be either $\hat{N}_j$ or $\hat{E}_j$ or $\hat{E}_j^\dagger$ as per Eq. (S7). The corresponding quantum field correlation function is gov-

erned by

$$C_s^{(1)}(\hat{f}_s,\hat{f}_a)=\langle\{\hat{f}_a,\hat{E}_a^\dagger(\tau)\}\rangle_a\langle[\hat{f}_s,\hat{E}_s^\dagger(t)]\rangle_s,$$
$$C_a^{(1)}(\hat{f}_s,\hat{f}_a)=\langle\{\hat{f}_s,\hat{E}_s^\dagger(\tau)\}\rangle_s\langle[\hat{f}_a,\hat{E}_a^\dagger(t)]\rangle_a. \qquad (S9)$$

This shows that the last interaction with the quantum field enters as a commutator, while the earlier interaction with the field is governed by the anti-commutator. Substituting individual terms in Eq. (S7) into these correlation functions and taking commutations and anti-commutations explicitly we obtain for the single molecule contribution to the total photon number at detector 1 as:

$$\langle\hat{N}_1(\omega_1,T)\rangle_1\propto\int_{-\infty}^{\infty}dt\int_{-\infty}^{t}d\tau\mathscr{E}_p(t)\mathscr{E}_p(\tau)g_{N1}^{(1)}(\omega_1,t,\tau), \qquad (S10)$$

where

$$g_{N1}^{(1)}(\omega,t,\tau)\propto$$
$$2A_a^*(\tau)\alpha_a^*(\alpha_{s'}A_{s'}(\omega_1)+\alpha_{a'}A_{a'}(\omega_1)e^{i\omega_1(t-T)}R_s(t,\tau)+$$
$$2A_s^*(\tau)\alpha_s^*(\alpha_{a'}A_{a'}(\omega_1)+\alpha_{s'}A_{s'}(\omega_1)e^{i\omega_1(t-T)}R_a(t,\tau)+c.c. \qquad (S11)$$

where we omitted envelope coefficients for brevity. Similarly one can obtain $\langle\hat{N}_2(\omega,T)\rangle_2$ by replacing $j\leftrightarrow j'$, $j=s,a$. we now turn to the two-molecule contributions which originate from $8\times8=64$ terms. The photon numbers $\langle\hat{N}_{1,2}(\omega,T)\rangle_2$ can be decomposed similarly to those for single molecule in Eq. (S7) but for two molecular case. The corresponding two-molecule correlation function for the quantum fields can be recast as

$$\langle\hat{f}_s^{(2)}(\hat{E}_s)\hat{f}_a^{(2)}(\hat{E}_a)\rangle_2\propto\int_{-\infty}^{\infty}dt\int_{-\infty}^{t}d\tau\int_{-\infty}^{\infty}dt'\int_{-\infty}^{t}d\tau'$$
$$\sum_{i,j=s,a}C_{ij}^{(2)}(\hat{f}_s^{(2)},\hat{f}_a^{(2)})\mathscr{E}_p(t)\mathscr{E}_p(\tau)\mathscr{E}_p^*(t')\mathscr{E}_p^*(\tau')R_i(t,\tau)R_j^*(t',\tau'), \qquad (S12)$$

where

$$C_{ss}^{(2)}(\hat{f}_s,\hat{f}_a)=\langle\{\hat{E}_a(\tau'),\{\hat{f}_a,\hat{E}_a^\dagger(\tau)\}\}\rangle_a\langle[\hat{E}_s(t'),[\hat{f}_s,\hat{E}_s^\dagger(t)]]\rangle_s,$$
$$C_{aa}^{(2)}(\hat{f}_s,\hat{f}_a)=\langle\{\hat{E}_s(\tau'),\{\hat{f}_s,\hat{E}_s^\dagger(\tau)\}\}\rangle_s\langle[\hat{E}_a(t'),[\hat{f}_a,\hat{E}_a^\dagger(t)]]\rangle_a,$$
$$C_{sa}^{(2)}(\hat{f}_s,\hat{f}_a)=\langle\{\hat{E}_s(\tau'),[\hat{f}_s,\hat{E}_s^\dagger(t)]\}\rangle_s\langle\{[\hat{E}_a(t'),\hat{f}_a],\hat{E}_a^\dagger(\tau)\}\rangle_a,$$
$$C_{as}^{(2)}(\hat{f}_s,\hat{f}_a)=\langle\{\hat{E}_a(\tau'),[\hat{f}_a,\hat{E}_a^\dagger(t)]\}\rangle_a\langle\{[\hat{E}_s(t'),\hat{f}_s],\hat{E}_s^\dagger(\tau)\}\rangle_s. \qquad (S13)$$

Note, that the quantum field, which interacts the last is entering via double commutator, while the field which interacts earlier enters via double anti-commutator. In the cross terms $\sim R_sR_a^*$ both fields contain both commutator and anti-commutator highlighting the quantum nature of the field which weighting different component of the nonlinear response differently. This special feature of the quantum treatment ensures that the $|R_j|^2$ terms have to have minimum two operators for the last field to have nonvanishing trace ($\hat{f}_j$ can be $\hat{N}_j$ and $\hat{N}_j^2$, but not $\hat{E}_j$ or $\hat{E}_j^\dagger$, $j=s,a$) while cross terms $\sim R_sR_a^*$ or $R_aR_s^*$ do not vanish for any number of field operators. This structure of the quantum field correlations is a sharp contrast with the classical case where all field contributions are identical and are given by a simple product of the field amplitudes. As the result we obtain

$$\langle\hat{N}_1(\omega_1,T)\rangle_2\propto\int_{-\infty}^{\infty}dt\int_{-\infty}^{t}d\tau\int_{-\infty}^{\infty}dt'\int_{-\infty}^{t}d\tau'C_p(t,t',\tau,\tau')$$
$$\times g_{N1}^{(2)}(\omega_1,t,t',\tau,\tau'), \qquad (S14)$$

where

$$g_{N1}^{(2)}(\omega_1,t,t',\tau,\tau')$$
$$\propto2(2N_{a0}A_a^*(\tau)A_a(\tau')+|A_a(\tau-\tau')|^2)e^{i\omega_1(t-t')}R_s(t,\tau)R_s^*(t',\tau')$$
$$+2(2N_{s0}A_s^*(\tau)A_s(\tau')+|A_s(\tau-\tau')|^2)e^{i\omega_1(t-t')}R_a(t,\tau)R_a^*(t',\tau')$$
$$+4\alpha_s^*\alpha_aA_s^*(\tau)A_a(\tau')e^{i\omega_1(t-t')}R_a(t,\tau)R_s^*(t',\tau')$$
$$+4\alpha_a^*\alpha_sA_a^*(\tau)A_s(\tau')e^{i\omega_1(t-t')}R_s(t,\tau)R_a^*(t',\tau'). \qquad (S15)$$

One can similarly obtain $\langle\hat{N}_2(\omega_2)\rangle_2$ by replacing $j\leftrightarrow j'$, $j=s,a$ and $\omega_1\leftrightarrow\omega_2$. We now turn to second order correlation functions:

$$Var(\hat{N}_1(\omega,T)-\hat{N}_2(\omega,T))_2=Var(\hat{N}_1(\omega,T))_2+Var(\hat{N}_2(\omega,T))_2$$
$$-Cov(\hat{N}_1(\omega,T),\hat{N}_2(\omega,T))_2-Cov(\hat{N}_2(\omega,T),\hat{N}_1(\omega,T))_2, \qquad (S16)$$

where each term can be expanded in terms of Stokes and anti-Stokes correlation functions. For instance for the first term we obtain:

$$Var(\hat{N}_1(\omega,T))_2=\langle\hat{N}_1^2(\omega,T)\rangle_2-2\langle\hat{N}_1\rangle_0\langle\hat{N}_1(\omega,T)\rangle_2$$
$$-\langle\hat{N}_1(\omega,T)\rangle_1^2, \qquad (S17)$$

where $\langle\hat{N}_1\rangle_0=N_{s0}+N_{a0}+\alpha_a^*\alpha_s+\alpha_s^*\alpha_a$ and $\langle\hat{N}_1(\omega,T)\rangle_{1,2}$ are given by Eq. (S10) and (S14), respectively. The first term in Eq. (S17) can be expanded as follows:

$$\langle\hat{N}_1^2\rangle_2=\langle\hat{N}_{s'}^2\rangle_2+\langle\hat{N}_{a'}^2\rangle_2+2\langle\hat{N}_{s'}\hat{N}_{a'}\rangle_2+\langle\hat{E}_{s'}^\dagger\hat{E}_{a'}\hat{E}_{a'}^\dagger\hat{E}_{s'}\rangle_2$$
$$+\langle\hat{E}_{a'}^\dagger\hat{E}_{s'}\hat{E}_{s'}^\dagger\hat{E}_{a'}\rangle_2 \qquad (S18)$$

Each of these correlation function can be calculated using Eq. (S13) and thus obtain

$$Var(\hat{N}_1(\omega_1,T))_2\propto\int_{-\infty}^{\infty}dt\int_{-\infty}^{t}d\tau\int_{-\infty}^{\infty}dt'\int_{-\infty}^{t}d\tau'C_p(t,t',\tau,\tau')$$
$$\times g_{Var(N_1)}^{(2)}(\omega_1,T,t,t',\tau,\tau'), \qquad (S19)$$

where

$$g^{(2)}_{Var(N_1)}(\omega_1,T,t,t',\tau,\tau') \propto 4e^{i\omega_1(t-t')}\{[(N_{a'0}|A_{a'}(\omega_1)|^2+N_{s'0}|A_{s'}(\omega_1)|^2)|A_a(\tau-\tau')|^2)+2N_{a0}A_a^*(\tau)A_a(\tau')$$
$$+2\alpha_{a'}^*\alpha_a A_{a'}^*(\omega_1)A_a(\tau')e^{i\omega_1(\tau-T)}+2\alpha_a^*\alpha_{a'}A_a^*(\tau)A_{a'}(\omega_1)e^{-i\omega_1(\tau'-T)}]R_s(t,\tau)R_s^*(t',\tau')$$
$$+[(N_{a'0}|A_{a'}(\omega_1)|^2+N_{s'0}|A_{s'}(\omega_1)|^2)|A_s(\tau-\tau')|^2)+2N_{s0}A_s^*(\tau)A_s(\tau')$$
$$+2\alpha_{s'}^*\alpha_s A_{s'}^*(\omega_1)A_s(\tau')e^{i\omega_1(\tau-T)}+2\alpha_s^*\alpha_{s'}A_s^*(\tau)A_{s'}(\omega_1)e^{-i\omega_1(\tau'-T)}]R_a(t,\tau)R_a^*(t',\tau')$$
$$+2\alpha_{s'}^*\alpha_s A_{s'}^*(\omega_1)A_s(\tau')e^{i\omega_1(\tau-T)}+2\alpha_a^*\alpha_{a'}A_a^*(\tau)A_{a'}(\omega_1)e^{-i\omega_1(\tau'-T)}]R_s(t,\tau)R_a^*(t',\tau')$$
$$+2\alpha_{a'}^*\alpha_a A_{a'}^*(\omega_1)A_a(\tau')e^{i\omega_1(\tau-T)}+2\alpha_s^*\alpha_{s'}A_s^*(\tau)A_{s'}(\omega_1)e^{-i\omega_1(\tau'-T)}]R_a(t,\tau)R_s^*(t',\tau')\}. \quad \text{(S20)}$$

One can similarly evaluate other terms in Eq. (S16) and finally obtain

$$Var(\hat{N}_1(\omega_1,T)-N_2(\omega,T))_2 \propto \int_{-\infty}^{\infty}dt\int_{-\infty}^{t}d\tau\int_{-\infty}^{\infty}dt'\int_{-\infty}^{t}d\tau' C_p(t,t',\tau,\tau')g^{(2)}_{Var(N_1-N2)}(\omega_1,T,t,t',\tau,\tau'), \quad \text{(S21)}$$

where

$$g^{(2)}_{Var(N_1-N_2)}(\omega,T,t,t',\tau,\tau') \propto 4e^{i\omega_1(t-t')}\{[(N_{a0}|A_{a-}(\omega)|^2+N_{s0}|A_{s-}(\omega)|^2)|A_a(\tau-\tau')|^2)+2N_{a0}A_{a-}^*(\tau)A_{a-}(\tau')$$
$$-2N_{a0}A_{a-}^*(\omega)A_{a-}(\tau')e^{i\omega(\tau-T)}-2N_{a0}A_{a-}^*(\tau)A_{a-}(\omega)e^{-i\omega(\tau'-T)}]R_s(t,\tau)R_s^*(t',\tau')$$
$$+[(N_{a0}|A_{a-}(\omega_1)|^2+N_{s0}|A_{s-}(\omega)|^2)|A_s(\tau-\tau')|^2)+2N_{s0}A_{s-}^*(\tau)A_{s-}(\tau')$$
$$-2N_{s0}A_{s-}^*(\omega)A_{s-}(\tau')e^{i\omega(\tau-T)}+2N_{s0}A_{s-}^*(\tau)A_{s-}(\omega)e^{-i\omega(\tau'-T)}]R_a(t,\tau)R_a^*(t',\tau')$$
$$-2N_{s0}A_{s-}^*(\omega)A_{s-}(\tau')e^{i\omega(\tau-T)}-2N_{a0}A_{a-}^*(\tau)A_{a-}(\omega)e^{-i\omega(\tau'-T)}]R_s(t,\tau)R_a^*(t',\tau')$$
$$-2N_{a0}A_{a-}^*(\omega)A_{a-}(\tau')e^{i\omega(\tau-T)}-2N_{s0}A_{s-}^*(\tau)A_{s-}(\omega)e^{-i\omega(\tau'-T)}]R_a(t,\tau)R_s^*(t',\tau')\}. \quad \text{(S22)}$$

where we assume $N_{s0}=N_{s'0}$ and $N_{a0}=N_{a'0}$ and $A_{j-}=A_j-A_{j'}$ is classical analogue of commutator ("-" superoperator).

The result in Eq. (S21) show that the final result depends only on commutators of quantum fields and therefore is zero when the two pair of quantum fields are exactly same. This result includes all pathways of R_s and R_a and the vanishing variance indicates the vanishing background. For a simple system with two vibrational modes and single reaction pathway (sequential energy transfer) it is sufficient to consider $\sim|R_s|^2$ term and therefore the second pair of quantum fields is not necessary. Therefore by setting e.g. $\hat{E}_s=\hat{E}_{a'}=0$ one can obtain the result in Eq. (4) of the main text (for clarity we omit "prime" in $E_{s'}$ in the main text.

S3. S3. GREEN'S FUNCTION FOR SLE

The basic SLE explanation is shown in Eq. (8) in the main text. Here we present the Green's function for the SLE for cyclobutane thymine for a vibrational mode ω_{ca}. Similar expressions can be done for ω_{da}. The coherent part $\hat{\mathscr{L}}_S=-(i/\hbar)[H_S,...]$ vanishes for the $|aa\rangle\rangle$ and $|cc\rangle\rangle$ blocks, $[\hat{\mathscr{L}}_S]_{aa,aa}=\hat{\mathscr{L}}_S]_{cc,cc}=0$. The remaining blocks are given by

$$[\hat{\mathscr{L}}_S]_{ac,ac}=i\begin{pmatrix}\omega_{ca1}&0&0&0\\0&\omega_{ca2}&0&0\\0&0&\omega_{ca3}&0\\0&0&0&\omega_{ca4}\end{pmatrix}. \quad \text{(S23)}$$

The two Liouville space Green's function in Eq. (4) are given by

$$\mathscr{G}_{aa}(t)\equiv\mathscr{G}_{aa,aa}(t)=-\frac{i}{\hbar}\theta(t)exp\left([\hat{L}_St]\right)$$
$$=-\frac{i}{\hbar}\theta(t)U\exp\left([\hat{L}_S]^{diag}t\right)U^{-1}, \quad \text{(S24)}$$

$$\mathscr{G}_{ac}(t)\equiv\mathscr{G}_{ac,ac}(t)=-\frac{i}{\hbar}\theta(t)exp\left([[\hat{L}_St]+[\hat{\mathscr{L}}_S]_{ac,ac}]t\right)$$
$$=-\frac{i}{\hbar}\theta(t)V\exp\left([\hat{\mathscr{L}}_S]^{diag}_{ac,ac}t\right)V^{-1}, \quad \text{(S25)}$$

where U and V are transformation matrices which diagonalize the matrices in the exponents. The corresponding response function is given by

$$R_s(t,\tau)\propto\sum_{a,c}\alpha_{ac}^2\langle\langle I|\mathscr{G}_{ac}(t-\tau)\mathscr{G}_{aa}(\tau)|\rho_0\rangle\rangle_S$$
$$\propto\sum_{a,c}\alpha_{ac}^2\theta(t-\tau)\theta(\tau)e^{-\gamma_a t}(1,1,1,1)$$
$$\times V\exp\left([\hat{\mathscr{L}}_S]^{diag}_{ac,ac}(t-\tau)\right)V^{-1}$$
$$\times U\exp\left([\hat{L}_S]^{diag}\tau\right)U^{-1}|\rho_0\rangle\rangle, \quad \text{(S26)}$$

where the initial state $|rho_0\rangle\rangle=|aa\rangle\rangle(1,0,0,0)$ and the final state is $\langle\langle I|=(1,1,1,1)(\langle\langle aa|+\langle\langle cc|)$. Vibrational dephasing has been added to the population Greens' function

$\mathscr{G}_{aa}$ as $e^{-2\gamma_a \tau}$ and to the coherence $\mathscr{G}_{ac}(t)$ as $e^{-\gamma_a (t-\tau)}$ where $\gamma_a^{-1} = 532$ fs.

[1] F Hong-Yi, Squeezed states: Operators for two types of one- and two-mode squeezing transformations. *Physical Review A* **41**, 1526 (1990).

[2] Z Ficek, R Tanaś, *Quantum-limit spectroscopy*. (Springer), (2017).

[3] DA Kopylov, T Meier, PR Sharapova, Theory of multimode squeezed light generation in lossy media. *Quantum* **9**, 1621 (2025).

[4] ME Tzur, et al., Generation of squeezed high-order harmonics. *Physical Review Research* **6**, 033079 (2024).
